# Effects of experimental immunosuppression in cattle with persistently high antibody levels to *Salmonella *Dublin lipopolysaccharide O-antigens

**DOI:** 10.1186/1746-6148-3-17

**Published:** 2007-08-07

**Authors:** Sanne R Lomborg, Jørgen S Agerholm, Asger L Jensen, Liza R Nielsen

**Affiliations:** 1 Dept. of Large Animal Sciences, Faculty of Life Sciences (LIFE), University of Copenhagen, Groennegaardsvej 8, DK-1870 Frederiksberg C, Denmark; 2 Dept. of Veterinary Pathobiology, LIFE, Ridebanevej 3, DK-1870 Frederiksberg C, Denmark; 3 Dept. of Small Animal Clinical Sciences, LIFE, Groennegaardsvej 3, st., DK-1870 Frederiksberg C, Denmark

## Abstract

**Background:**

*Salmonella *Dublin (*S*. Dublin) is a zoonotic bacterium which is host adapted to cattle. The bacterium can cause subclinical persistent infection in cattle (carriers), which may be reactivated. During reactivation, animals may shed bacteria, thus constituting a source of infection for other animals. Identification of such carriers is assumed to be critical in attempts to control and eradicate the infection. Some authors suggest that persistently high antibody levels in serum or milk is indicative of a carrier state in cattle. However, this has been questioned by other studies in which *S*. Dublin were not found in all animals suspected of being carriers based on antibody measurements when such animals were examined at slaughter. Some hypothesize that the lack of isolated bacteria from long-term high antibody level cattle is due to a latent infection stage that can later be reactivated, for instance during stress around calving or due to transportation.

This study examined nine adult cattle with persistently high antibody responses to *S*. Dublin O-antigen based lipopolysaccharide for cultivable bacteria in faeces, milk and internal organs before and after transportation, isolation and experimental immunosuppression with dexamethasone sodium phosphate over a period of 7–14 days.

**Results:**

Clear signs of immunosuppression were seen as expression of leucocytosis and neutrophilia in all animals on day 3–5 after the first injections with dexamethasone sodium phosphate. No clinical signs or necropsy findings indicating salmonellosis were observed in any of the animals. No shedding of *S*. Dublin was found in faeces (collected four times daily) or milk (collected twice daily) at any point in time during the 7–14 day period. *S*. Dublin was recovered by a conventional culture method from tissue samples from mammary lymph nodes, spleen and liver collected from three animals at necropsy.

**Conclusion:**

In this study, immunosuppression by transportation stress or dexamethasone treatment did not lead to excretion of *S*. Dublin in milk or faeces from infected animals. The study questions the general conception that cattle with persistently high antibody levels against *S*. Dublin O-antigens in naturally infected herds should be considered high risk for transmission and therefore culled as part of effective intervention strategies. It is suggested that the location of *S*. Dublin infected foci in the animal plays a major role for the risk of excreting bacteria.

## Background

*Salmonella enterica *subsp. *enterica *serovar Dublin (*S*. Dublin) is a zoonotic bacterium which is host adapted to cattle. Although it infects cattle at all ages, severe clinical disease is mostly seen in calves [1]. The bacterium occasionally infects humans where it causes severe illness and high case mortality due to septicaemia [2].

An epidemiologically important feature of *S*. Dublin is its ability to cause subclinical persistent infection in cattle (carriers) [3]. Such carriers probably harbour the bacterium in cells of the reticular-endothelial system such as the liver and spleen [4] and it is assumed that reactivation of the infection can occur [3,5,6]. It has been hypothesized that reactivation may be caused by stress due to transport or immunosuppression [7-9]. During reactivation animals may shed bacteria and contaminate the environment, thus constituting a source of infection for other animals [10]. Identification of such carriers is assumed to be critical in attempts to control and eradicate the infection [11-14].

Bacteriological culture is a common method to diagnose salmonellosis, but due to intermittent shedding of bacteria in milk and faeces by carrier animals, sensitivity of conventional bacteriological culturing is poor in such animals [11,15]. However, serological analyses have indicated that carrier animals elicit a more persistent antibody response to *S*. Dublin lipopolysaccharide (LPS) than recently infected animals that have eliminated the infection [11,13,16,17]. This has formed the basis for recommendations for control of *S*. Dublin, i.e. identifying carriers by demonstration of persistently high antibody levels against *S*. Dublin LPS by ELISA on blood or milk [12,14]. The positive predictive value of the test is, however, questionable, meaning that not all animals detected as carriers based on antibodies are truly infected. It has been shown that the bacterium can be isolated at slaughter from around 50% of such persistently seropositive cattle [18]. A low positive predictive value has negative economic implications for the producers, because productive animals may be culled at disadvantageous times. On the other hand, a low negative predictive value would allow for undesired and unknown transmission of infection in the face of a test-and-cull strategy for handling of carrier animals.

Effective and cost efficient eradication of *S*. Dublin infections in cattle requires detailed knowledge about the pathogenesis of persistent *S*. Dublin infection, including risk assessment on animals with persistently high antibody titres, and the availability of tests with high predictive values for large scale screenings. The aim of this study was to evaluate if reactivation of a latent infection with *S*. Dublin occurs following transportation and immunosuppression in naturally infected cows with persistently high antibody responses to *S*. Dublin O-antigen based LPS. The study also adds further knowledge to the distribution of *S*. Dublin bacteria in tissues of cows with persistently high antibody responses.

## Results and discussion

### Antibody levels

**Figure 1:**
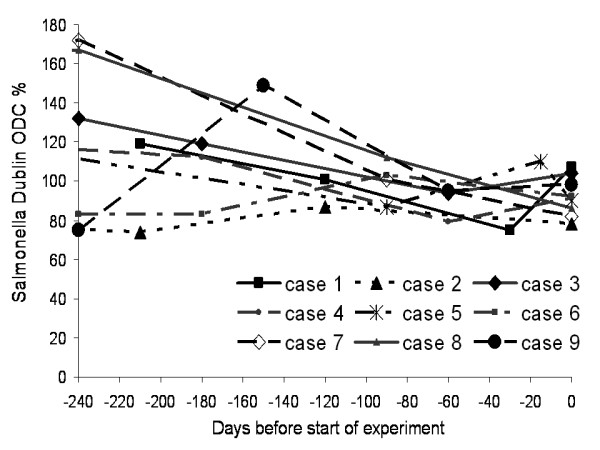
Repeated antibody measurements in nine cattle suspected as *Salmonella *Dublin carrier animals at study start (day 0) and during the preceding 240 days. ODC% indicates the level of antibodies to *Salmonella *Dublin O-antigen based LPS in serum or milk.

Nine animals from four dairy herds were included in the study. The antibody levels (*S*. Dublin ODC%) at time of arrival were above 80 for 8 animals, while it was 78 for one cow (No. 2). The ODC% had either slightly decreased or had remained at approximately the same level through the preceding 240 days (Figure 1).

### Clinical symptoms

The animals were of normal condition at arrival. During the study period mild symptoms related to localised *Sarcoptes *mange (No. 2), chronic mastitis or positive California Mastitis Test (Nos. 1–6) and traumatic injuries of the distal parts of the legs (No. 2) were observed. The most frequent abnormal clinical finding was reduced appetite (four cows). Furthermore, two cows had reduced rumen motility. Diarrhoea occurred in two animals (Nos. 4 and 8) after treatment with dexamethasone sodium phosphate (DSP). Cases Nos. 1 and 8 aborted on day 5.

### Haematology

**Figure 2:**
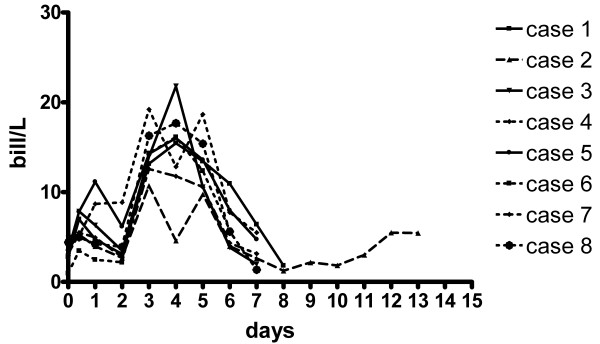
Measurements of segmented neutrophil granulocytes (SNG) in eight cattle with persistently high antibody levels to *Salmonella *Dublin. The animals were injected with dexamethasone sodium phosphate on day 2, 3 and 4 (reference values: 0.60–5.65 billions SNG/L).

The haematological profiles were within the normal range from day 0 through 3, but a slight increase in the number of segmented neutrophilic granulocytes (SNG) occurred on day 0. On day 3 a marked increase in SNG due to leucocytosis and neutrophilia was observed (Figure 2).

### Necropsy

Necropsy revealed a range of lesions of which some were incidental findings as chronic multifocal interstitial nephritis (No. 3), and chronic mastitis (Nos. 3 and 6), while others were most likely related to the relocation of animals and DSP treatment (acute haemorrhagic abomasitis predominantly at the margins of the abomasal plicae (Nos. 1, 3, 4, 6, 7) and diffuse low-grade hepatic steathosis (No. 6)). Four cows had rumenitis probably due to rumenal acidosis before or during the study (Nos. 2, 3, 5, 9).

### Bacteriological culture

Bacteriological culture of milk and faeces samples collected throughout the study failed to demonstrate any excretion of *Salmonella *sp. Bacteriological examination of 14–16 organ specimens from each animal sampled at necropsy revealed the presence of *S*. Dublin in three animals originating from one herd. *S*. Dublin was isolated from mammary lymph node and the liver of cows No. 3 and 7 respectively, while it was isolated from both the liver and spleen of No. 8. The aborted foetus of cow No. 8 was not examined. No other serotypes of *Salmonella *were found.

Cattle with persistently high antibody responses to *S*. Dublin LPS are expected to harbour the bacterium and control strategies for *S*. Dublin are often based on this hypothesis [8,13,17]. We were only able to isolate *S*. Dublin from three out of eight cows and one heifer even though all fell within the detection criteria for carriers. This may be due to an insufficient sensitivity of bacteriological examination, but it may also reflect a complete elimination of bacteria by previously infected animals, in particular in adult cows. A previous study in Danish cattle demonstrated *S*. Dublin in 2 of 14 (14%) persistently seropositive adult cows at necropsy despite extensive tissue culturing, while 10 of 17 (59%) of the young cattle (heifers and bulls) were culture positive at necropsy [18]. The animals all came from herds with recent clinical outbreaks of *S*. Dublin and no control strategies in place. In another study, 3 of 8 persistently seropositive adult cattle were culture positive at necropsy from animals that had *S*. Dublin isolated from both milk or faeces during the preceding six months indicating active infections prior to necropsy [11]. They all came from the same large dairy herd which suffered from severe *Salmonella *Dublin related clinical problems and the herd used vaccination of cows as part of the control strategies. Thus, the study group in that study was not comparable to the cattle used in the present study. The persistent discrepancy between the serological and bacteriological findings may indicate that only few cows with long-term high antibody responses are truly infected with *S*. Dublin, thus making antibody based testing unreliable in adult cattle. The present study adds knowledge about persistently seropositive cows in herds that have performed control strategies for an extended period of time. These cows continued to be seropositive even under circumstances where herd mates had decreasing or low antibody titers to *S*. Dublin. It could be postulated that some cows with a strong antibody response in fact could be desirable, as they may reflect a superior capability to eliminate *S*. Dublin.

It is likely that excretion of bacteria in faeces and milk reflects the localisation of the infection in the gastrointestinal tract or the mammary gland, or a temporary infection from the environment. It is obvious that a surface lesion such as an intestinal mucosal ulceration is more likely to release bacteria to the environment than a lesion in internal organs such as the spleen. *S*. Dublin was not found in faeces of the cattle in this study, neither before nor after immunosuppression. In accordance with this, bacteria were not isolated from intestinal tissue or intestinal lymph nodes at necropsy. It is likely that the animals in this study did not have infectious foci in the intestinal tract. It is possible, that the culture negative results in faeces found in other studies may have a similar explanation and that discrepancy between culture positive faeces samples and culture negative intestinal tissue specimens may be due to passive transfer of orally acquired bacteria in the intestinal content without mucosal colonisation.

The detection of *S*. Dublin in the liver and spleen probably reflects a previous haematogenous spread. This may either have been restricted to the portal circulation with localisation only in the liver or have been systemic with localisation in multiple tissues. *S*. Dublin was only isolated from a mammary lymph node in one cow. This may reflect a previous *S*. Dublin associated mastitis, but it is more likely that the lymph node localisation is associated with a previous bacteraemia with a primary location in the lymph node or secondary to a localisation in the drainage area.

An important epidemiological aspect of bovine salmonellosis may be the reactivation and excretion of bacteria following stress and immune suppression [5,7-9]. Reactivation apparently occurs rapidly. In an experimental study faecal excretion in carrier animals happened within one day after transportation [5,9]. Therefore, a study period of 7 to 14 days was considered sufficient to detect reactivation if it occurred. The animals in this study were transported, relocated into isolation facilities and finally treated with DSP to induce immunodepression and subsequent reactivation of a latent infection. These events led to changes in the haematological profiles (Figure 2) consistent with DSP induced immune suppression, but reactivation of the infection apparently did not occur. This may be explained by the location of bacteria in the actual animals combined with the mechanisms by which DSP induces immunosuppression. DSP induces a pronounced down regulation of surface L-selectin on circulating neutrophils, which impede their ability to adhere to the endothelium. Consequently, their migration into tissues is hindered and extravascular immunodepression develops [19]. If it is assumed that bacteriological examination identified the only infected tissues (liver, spleen, and lymph node) then excretion of bacteria in milk or faeces would require haematogenous spread to the intestine and mammary gland. Thus, even if the bacteria were reactivated, they had to enter the blood to reach these organs. Since DSP does not impede the killing mechanisms of neutrophils [20,21], bacteria entering the blood probably would have been eliminated. The situation is likely to be different in animals harbouring *S*. Dublin in tissues with an external surface such as the intestine, tonsils and mammary gland. DSP treatment of such animals may reactivate the infection, and because haematogenous spread is not needed, such animals may release bacteria. This hypothesis is in accordance with the observations by Spier et al. (1991) [8] who observed a significant increase in excretion of *S*. Dublin in milk of udder infected cattle following DSP treatment. In the experimental studies of the effect of transportation on shedding patterns in cattle, it is quite possible that the animals had bacteria in the gut even when they were not shedding before exposed to the stressors [5,9] therefore making it easy to return to a shedding state after transportation. Our study indicates that true reactivation of a latent infection located in organs outside external surfaces is not likely to occur under farm conditions.

## Conclusion

This study raises several questions regarding the use of repeated antibody measurements for detection of cows persistently infected with *S*. Dublin and the pathogenesis of reactivation. The isolation of *S*. Dublin from only 2 of 8 adult animals despite intensive bacteriological culturing of both faecal matter, milk and target organs questions the reliability of both serology and bacteriology in this age group. It is important to have reliable diagnostic tools for identification of persistently infected animals to control the spread of the infection within and between herds efficiently, and the study emphasises these needs.

Though the sample size is not large, the study also indicates that the risk of excretion of bacteria may depend on the localisation of infectious foci. It is likely that cattle harbouring *S*. Dublin in organs without an external surface are of lower risk of releasing bacteria to the environment than animals with intestinal or mammary infections. Thus, reactivation and excretion of bacteria is probably not only a matter of immunosuppression and latent infection but mainly a question of tissue localisation.

Finally, it is possible that other mechanisms of immunosuppression different from those of DSP may lead to reactivation of latent infection, but the pathogenesis of such a mechanism in relation to *Salmonella *infection and reactivation remains to be described. In order to better understand the mechanisms, it appears to be important to differentiate between true latent infections and active persistent infections with continuous or intermittent shedding.

## Methods

### Animals

Eight lactating Holstein cows (cases 1 – 7 and 9) and one Holstein heifer (case no. 8) (age range 1 1/2 – 6 years, average: 4 years) of which three were pregnant were included in the study. The animals originated from four herds tested free for bovine virus diarrhoea virus and assumed to be free of several pathogens including bovine herpesvirus type 1, bovine leucosis virus, *Brucella abortus*, and *Mycobacterium bovis *due to the national disease status. The herds had naturally acquired infection with *S*. Dublin and participated in a *S*. Dublinintervention project. The animals were selected for the study based on antibody levels to *S*. Dublin LPS of at least 80 ODC % (background-corrected ratio of the optical density to a positive reference) during a period of at least 180 days measured by ELISA in three individual milk or blood samples collected every three months (Figure 1). While other cows in the herds had decreasing antibody levels these nine animals remained high in antibody levels for at least 180 days.

### Experimental design

The animals were transported two at a time by truck for 4–6 hours from four herds of origin and were housed separately in isolation facilities at the research institution. The animals were separated by a wooden wall during transportation. The animals were examined clinically before they left the herd of origin and blood, milk and faeces were sampled (day 0). Additional blood, milk and faecal samples for serology, bacteriology or haematology were collected upon arrival at the isolation facility. Clinical examinations and sampling for bacteriological culture and serology were performed daily. The animals were treated with dexamethasone sodium phosphate (DSP) at a dose of 0.08 mg/kg intramuscularly (Dexadreson^® ^Vet, Intervet International, Boxmeer, The Netherlands) on day 2, 3 and 4. The animals were euthanized by intravenous injection of a sodium pentobarbital solution seven to 14 days (average 7.8 days) after arrival and necropsied. Samples for bacteriology and histopathology were taken at necropsy. Case no. 9 was only included in a part of the study as it was euthanized on day one due to accidental injuries.

### Housing and Management

The animals were housed separately in isolation facilities. Only two animals were allowed in the barn in the same period and they were housed alone in each of two fully closed isolation rooms. All daily routines were structured in a way to minimize the risk of cross contamination between two animals in adjacent isolation rooms. The animals were fed 4–6 kg concentrated feed twice daily and had free access to grass silage and water. Milking was done twice daily in lactating animals. The study was performed during a three-month-period with ambient outdoors temperature of 7 to 20°C.

### Clinical examination

Full clinical examination was performed daily, while rectal temperature, respiratory and pulse rates were recorded twice daily.

### Bacteriology

Milk samples (50 ml) of lactating animals were collected aseptically to avoid faecal contamination from all functional quarters twice a day and pooled, while faeces (50 g) was sampled rectally four times daily. The samples were stored at 4°C until analysis, which was started within 24 hours, except for samples taken on Fridays and Saturdays, which were stored for around 72 and 48 hours before analysis, respectively.

Tissue samples were taken at necropsy. Whenever possible, 25 g of tissue was sampled. The tissues included tonsils, lung, tracheobronchial lymph nodes, spleen, liver, liver lymph nodes, gall bladder, ileum, colon, colon lymph node, gut associated lymphoid tissue of colon (colon tonsil), cecum, cecal lymph node, uterus including placentomes if present, mammary gland and mammary lymph nodes. Lung, liver, gall bladder and abomasal content of an aborted foetus were also examined. Instruments were disinfected in 96% ethanol between each tissue sample.

The samples were cultured by conventional culturing as previously reported [22]. In short, Buffered Peptone Water (BPW, Merck 1.07728) were added to 25 g sample to a dilution of 1:10 and incubated over night at 37°C. From the pre-enriched buffer, inoculation was performed on two enrichment medias: a) 100 ml onto Modified Semi-solid Rappaport Vassiliadis (MSRV, Oxoid CM910); b) 1 ml in 9 ml selenite cysteine broth (SC, Merck 1.07709). Both mediawere incubated at 41.5°C for 24 hours. Culture negative plates were incubated for further 24 hrs. After enrichment, 10 ml from the broths and material from swarming zones of probably positive MSRV-plant were inoculated in parallel onto Brilliant Green Agar (BGA, OxoidCM 329) and Xylose Lysine Deoxycholate agar (XLD, Oxoid CM469). These plates were incubated at 37°C for 24 hrs. Isolated strains were verified by serotyping according to the Kauffman-White scheme.

### Serology

Daily blood samples were collected from the jugular or caudal vein in unstabilised vacutainer tubes. The samples were centrifuged at 2,000 *g *at 4°C for 10 minutes after coagulation, which occurred during five to 24 hours of storage at 4°C. Serum was aspirated and stored in cryotubes at -18°C until analysis.

The level of antibodies to *S*. Dublin LPS was analysed at Steins Laboratory, Holstebro, Denmark as previously described [22]. In short, analyses were performed by an indirect O-antigen based LPS serum- or milk-ELISA. Results were measured in optical density (OD) by an ELISA plate reader. The observed OD was corrected for background OD using negative test sera and expressed as ODC%:

ODC% = (OD_sample _- OD_neg ref_)/(OD_pos ref _- OD_neg ref_) × 100%

where OD_sample _= mean OD of the two test sample wells, OD_neg ref _= mean OD of the four negative reference sample wells, and OD_pos ref _= mean OD of the four positive reference test sample wells.

### Haematology

Daily blood samples were collected as for serology in 10 ml EDTA-stabilised vacutainers for haematological profiles. Analysis was performed within the same day except for samples taking during weekends, which were stored at 4°C until examination.

The samples were analysed by automatic flow cytometry using an automated analyser (ADVIA120, Bayer) with species-specific software. Automatic differential cell counts of leucocytes were carried out and histograms were visually inspected as were blood smears to evaluate if automatic counting was correct. If doubt arose, manual differential cell counts were performed on 100 leukocytes.

### Histopathology

The animals were necropsied and evaluated for gross lesions. Lesions were sampled, fixed in 10% neutral buffered formalin, and processed for microscopy by routine histological methods. Tissue sections were stained by haematoxylin and eosin.

## Authors' contributions

SRL: Study design, experimental work, laboratory and data analysis, preparation of manuscript, JSA: Study design, experimental work, preparation of manuscript.  ALJ: Laboratory analyses, preparation of manuscript, LRN: Study design, experimental work, data analysis, preparation of manuscript. All authors read and approved the final manuscript.
